# Authors’ reply

**DOI:** 10.4103/0971-9261.70651

**Published:** 2010

**Authors:** Umar Amin Qureshi, Nisar Ahmad, Akhter Rasool, Suhail Choh

**Affiliations:** Department of Neonatology and Pediatrics, SKIMS, Srinagar, Kashmir, India; 1 Department of Radiodiagnosis, SKIMS, Srinagar, Kashmir, India

Sir,

In response to the above query, the authors would like to mention that the cause of indirect hyperbilirubinemia was investigated on the lines of hemolytic anemia (peripheral blood film – spherocytosis; G6PD levels – G6PD deficiency), sepsis (hemogram, C-reactive protein, blood culture), urinary tract infection (urine microscopy and culture), hypothyroidism (thyroid function tests) and galactosemia (urine-reducing substance). Investigations were normal. Breast feeding was stopped for 24 h to rule out breast milk jaundice. Screening for rare metabolic disorders was not pursued once diagnosis of adrenal hemorrhage, a known cause of neonatal jaundice, was confirmed.

